# Sodium valproate prescribing safety in women of childbearing potential

**DOI:** 10.1192/bjo.2021.243

**Published:** 2021-06-18

**Authors:** Shane Donnelly, Rajesh Alex

**Affiliations:** Bassetlaw Mental Health Services

## Abstract

**Aims:**

The purpose of this audit was to identify all women being prescribed Sodium Valproate under the Bassetlaw Local Mental Health Team (LMHT) caseload to see how well latest prescribing guidelines are being met, and help set up a system allowing efficient monitoring of Sodium Valproate prescribing in the future.

**Background:**

Despite early concerns regarding potential teratogenicity, Sodium Valproate became a widely used anticonvulsant and mood stabiliser and is licensed for use in Epilepsy, Migraine prophylaxis and Bipolar affective disorder. Research evidence now shows its use in pregnancy increases risk of neurodevelopmental disorders to 40%, and serious birth defects to 10%. Despite research finding these risks prescribing practice did not significantly change. To better reflect these findings in clinical practice in 2018 the Pharmacovigilance Risk Assessment Committee recommended Sodium Valproate should not be used in pregnancy unless they have a form of epilepsy unresponsive to other anti-epileptic drugs, and all with childbearing potential should be enrolled in a pregnancy prevention programme (PPP). This was endorsed by UK Medicines and Healthcare Devices Regulatory Agency in April 2018 with launch of the PPP.

Standards:

Must be offered counselling about risks of valproate to unborn child and importance of effective contraception.

Annual specialist Review by a specialist now mandatory

Risk acknowledgement form must be updated at least annually.

**Method:**

The electronic RiO records for all female patients on the Bassetlaw LMHT caseload in the year 2019 were checked to identify those prescribed Valproate. For those prescribed Valproate, evidence of annual risk acknowledgement form, date of last appointment, underlying diagnosis and contraceptive method was checked. This data was stored together on an excel file and used to create a patient list to help allow future monitoring.

**Result:**

From 594 female patients identified, 27 (4.5%) were prescribed Sodium Valproate. Of these, 14 (52%) had PPP documentation uploaded, 24 (89%) had been reviewed within the last 12 months, and 13 (48%) had no documentation of contraceptive method.

**Conclusion:**

This audit helped highlight there is likely a large population of patients not yet on the Pregnancy Prevention Programme. Creating a monitoring system in excel for female patients being prescribed Valproate can help improve adherence to latest guidelines, with a colour coding system to highlight those needing risk acknowledgement forms/appointments within the next three or six months. Educating patients and other healthcare professionals about risks will also help improve prescribing practice and avoid use in pregnancy.

